# Base editing of Artemis mutations ex vivo sheds light on gene therapy for Artemis-deficient SCID

**DOI:** 10.1007/s44307-026-00115-w

**Published:** 2026-05-15

**Authors:** Ziwen Huang, Zhenxi Cai, Peiyang Yan, Yiheng Hu, Ying Wang, Gaijing Zhou, Lisi Deng, Xinli Wang, Zhen Li, Weiting Chen, Xin Tao, Anlong Xu, Shaochun Yuan

**Affiliations:** 1 SYSU Institute of Advanced Studies Hong Kong, Science Park, Hong Kong, 999077 People’s Republic of China; 2 Guangdong Provincial Key Laboratory of Pharmaceutical Functional Genes, MOE Key Laboratory of Gene Function and Regulation, MOE Engineering Center of South China Sea Marine Biotechnology, State Key Laboratory of Biocontrol, School of Life Sciences, Sun Yat-Sen University, Guangzhou, 510275 People’s Republic of China; 3 Conservation and Utilization Laboratory of Mountain Characteristic Resources in Guangdong Province, School of Life Sciences, Jiaying University, Meizhou, 514015 People’s Republic of China; 4 School of Medicine, Shenzhen Campus of Sun Yat-Sen University, Sun Yat-Sen University, Shenzhen, 518107 People’s Republic of China

**Keywords:** Artemis, ART-SCID, Gene editing, CBE, ABE

## Abstract

Artemis is an endonuclease that cleaves DNA hairpins during V(D)J recombination, a critical step enabling coding-joint formation and essential for lymphocyte development. Artemis-deficient severe combined immunodeficiency (ART-SCID) is a monogenic disorder marked by impaired lymphocyte development and poor responsiveness to allogeneic hematopoietic stem cell transplantation. In this study, we explored the use of base editors to correct mutations in the *Artemis* gene. Specifically, we firstly documented the pathogenic mutations of *Artemis* from previous studies and newly identified from ClinVar database. Then, we performed ex vivo assays using cytidine base editors (CBEs) to repair the c.181T > C mutation, and adenosine base editors (ABEs) to target the pathogenic c.49G > A and c.404G > A variants associated with ART-SCID. Targeted deep sequencing revealed that rAPOBEC1-SpRY-HF1-BE4max achieved efficient editing (~ 50%) at the c.181T > C site, while ABE8e reached ~ 35% and ~ 20% editing efficiency at the c.49G > A and c.404G > A sites, respectively. Importantly, base editors restored Artemis endonuclease activity in a 293T^*Artemis*−/−^ system, with no detectable off-target effects at the predicted sites as assessed by targeted deep sequencing. These findings provide proof-of-concept that base editing can correct ART-SCID-associated mutations, highlighting its potential for future therapeutic development.

**Supplementary Information:**

The online version contains supplementary material available at 10.1007/s44307-026-00115-w.

## Introduction

Severe combined immunodeficiency (SCID) is a group of inherited disorders characterized by impaired lymphocyte development (Fischer et al. 2015). To date, mutations in more than 20 genes have been implicated in SCID, such as *CD3D*, *IL7R*, *IL2RG*, *RAG1*, *RAG2*, *PRKDC*, *LIG4*, and *DCLRE1C* (also known as *Artemis*) (Aranda et al. 2024). SCID infants typically present with symptoms such as chronic diarrhea, oral thrush, skin rashes, and recurrent, refractory infections. Without early diagnosis and intervention, SCID can lead to life-threatening complications and a high risk of mortality.

Since 1960s, hematopoietic cell transplantation (HCT) has become the primary treatment for SCID patients, and thousands of infants have been treated by HCT around the world (Slatter and Gennery 2022). However, for the Artemis-deficient SCID (ART-SCID) patients, HCT treatment harbors some challenges. Artemis is an endonuclease that resolves the DNA hairpin intermediates during the V(D)J recombination process, which is crucial for the development of lymphocytes (Moshous et al. 2001). After RAG1/2 recombinase complex cleaves the recombination signal sequences (RSS), Artemis, activated by DNA-PKcs, nicks the closed DNA hairpin coding ends, creating a 3′ overhang that primes subsequent junctional diversification of antigen-receptor repertoire (Ma et al. 2002). It also acts as a caretaker of genomic integrity by participating in the non-homologous end-joining (NHEJ) DNA repair pathway (Dudasova and Chovanec 2003). Deficiency of Artemis results in arrest of T and B cell development, and leads to a radiation-sensitive form of SCID (RS-SCID) (Moshous et al. 2001). The genetic and clinical spectrum associated with *Artemis* mutations is highly heterogeneous. While null mutations, such as deletions, nonsense, frameshift, or disruptive splice variants, generally result in classical SCID with arrest of lymphocyte development, hypomorphic alleles retaining partial nuclease activity can give rise to atypical phenotypes, including Omenn syndrome and leaky SCID (Felgentreff et al. 2015; Pannicke et al. 2010). From the clinical investigations, RS-SCID patients are systemic sensitivity to alkylating agents, the standard pre-HCT myeloablative drugs used to eradicate recipient hematopoietic stem cells (HSCs) (O'Marcaigh et al. 2001; Wang et al. 2005). Clinical studies also indicated that ART-SCID patients remain hypersensitive to ionizing radiation and suffer more infections long after allogeneic HCT (Schuetz et al. 2014). Even with an HLA-matched sibling, the T and B cells reconstitution in ART-SCID patients after HCT treatment is not as efficient as it does in other SCID genotypes (Haddad et al. 2018; O'Marcaigh et al. 2001). Hence, alternative therapeutic avenues beyond HCT are demanded for ART-SCID patients.

To treat Artemis mutations, gene delivery strategies using a lentiviral vector that carries the wild-type *Artemis* cDNA driven by the strong human elongation factor-1α (EF1α) promoter was employed (Charrier et al. 2019; Multhaup et al. 2015). This configuration restored gene expression, yet resulted in a dose-dependent cytotoxic response (Multhaup et al. 2010). Although replacing the EF1α promoter with the endogenous human Artemis promoter produced near-physiological expression and achieved robust immune recovery in affected infants after in-vivo transduction of HSCs (Cowan et al. 2022; Multhaup et al. 2011; Punwani et al. 2017), the safety concern of insertional mutagenesis is lingering. Replication-competent retroviruses or lentiviruses (RCRs/RCLs) vector integrates semi-randomly, and their persistent replication may lead to proviral insertions into host genome, progressively increasing the risk of oncogene activation in patients (Milone and O'Doherty 2018). Besides gene delivery strategies, CRISPR-Cas9 mediated homology directed repair (HDR) offers another therapeutic approach for gene correction in HSCs (Pavel-Dinu et al. 2019). However, its clinical application also remains challenging, as HDR repair requires co-delivery of donor DNA templates into HSCs. Moreover, quiescent HSCs are intrinsically resistant to HDR, and the error-prone NHEJ pathway competes for HDR repair, collectively resulting in the low efficiency of HDR in HSCs (Shin et al. 2020).

In the past decade, gene editing technologies are advancing rapidly and have demonstrated immense therapeutic potential for genetic diseases (Chai et al. 2023; McAuley et al. 2023). Among the rapidly evolving gene-editing platforms, cytosine base editors (CBEs) and adenine base editors (ABEs) have emerged as the two dominant tools. Both of CBE and ABE consist of an engineered deaminase fused to a Cas9-derived module for sgRNA-guided DNA targeting. CBEs employ the cytidine deaminases, such as rat APOBEC1, to convert C•G to T•A via a C-to-U intermediate, while ABEs use evolved adenine deaminases (e.g., TadA*) to convert A•T to G•C through A-to-I deamination (Gaudelli et al. 2017; Komor et al. 2016). Base editors have been employed in clinical trials targeting diseases such as sickle cell disease (SCD), β-thalassemia, and familial hypercholesterolemia, as well as in the development of universal chimeric antigen receptor (CAR) T cells (Jiang and Yang 2023). Leveraging rapidly advancing base-editing precision, McAuley et al. delivered CBE by electroporation into human HSCs to correct the *CD3D* mutation that causes SCID, achieving approximately 71.2% pathogenic-allele repair (McAuley et al. 2023). To test the therapeutic potential of ART-SCID by base editing, here we first summarized the *Artemis* associated mutation driving SCID, and deployed the latest CBEs and ABEs tools to correct the c.49G > A, c.181T > C, and c.404G > A mutations, that severely impair Artemis activity. Ex vivo editing achieved 50% for c.181T > C and 30% for c.49G > A, restoring Artemis function and validating base editors as a potent therapeutic avenue for ART-SCID.

## Results

### Summarization and identification of the pathogenic mutations in *Artemis* gene

To explore the therapeutic potential of base editing for ART-SCID, we first summarized previously reported pathogenic point mutations in Artemis (Cowan et al. 2022; de Villartay et al. 2009; Ege et al. 2005; Evans et al. 2006; Felgentreff et al. 2015; Hazar et al. 2024; Huang et al. 2025; Inoue et al. 2023; Lagresle-Peyrou et al. 2008; Le Deist et al. 2004; Lee et al. 2013; Li et al. 2002; Ma et al. 2002; Moshous et al. 2001; Mou et al. 2021; Musio et al. 2005; Noordzij et al. 2003; Pannicke et al. 2010; Sundin et al. 2019; van der Burg et al. 2007; Woodbine et al. 2010) (Table S1). These mutations include D17N, H35D, D37G, D136N, D165N, H228N, and H254L, which contribute to the catalytic core of Artemis (Karim et al. 2020).

**Fig. 1:**
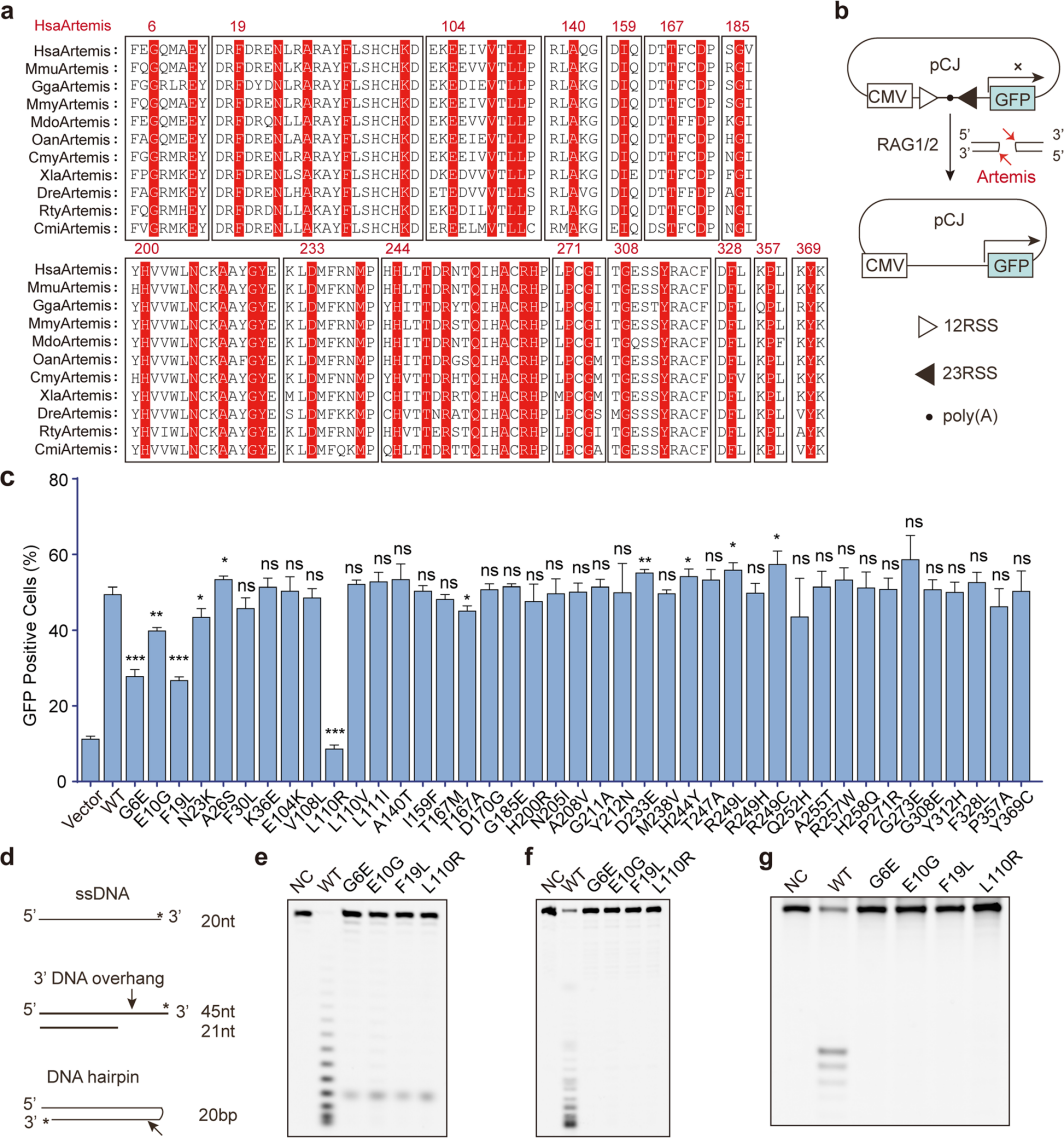
Four Artemis mutations were identified to impair its nuclease activities. **a** Evolutionarily conserved residues in Artemis proteins. Hsa, *Homo sapiens*; Mmu, *Mus musculus*; Gga, *Gallus gallus*; Mmy, *Myotis myotis*; Mdo, *Monodelphis domestica*; Oan, *Ornithorhynchus anatinus*; Cmy, *Chelonia mydas*; Xla, *Xenopus laevis*; Dre, *Danio rerio*; Rty, *Rhincodon typus*; Cmi, *Callorhinchus milii*. **b** Artemis endonuclease activity was assessed in Artemis-deficient 293T (293T^*Artemis*–/–^) cells using a coding joint (CJ) reporter assay. **c** The CJ reporter assays demonstrated that the G6E, E10G, F19L, and L110R mutations significantly impair Artemis activity compared with the wild-type protein. **d** Three DNA substrates used to evaluate Artemis nuclease activity. Asterisk indicates Cy5 labeling. **e**—**g** All four Artemis mutations reduce its nuclease activity toward single-stranded DNA (**e**), 3′ DNA overhangs (**f**), and DNA hairpin substrates (**g**). Flow cytometry results are presented as mean ± SD from three biological replicates. *P* values were calculated using Student’s t-test. ns: Not significant. In vitro experiments were conducted in three independent replicates, and one representative result is presented

To gain deeper insights into the association between Artemis mutations and disease, and to systematically identify suitable sites for gene editing, we further conducted an additional analysis of Artemis-related variants deposited in the ClinVar database. Then, 41 mutations documented as disease-associated mutations in the ClinVar database and highly conserved throughout Artemis evolution, were finally filtered out for further functional characterization (Fig. 1a, Table S1). Then, the previously established 293T^*Artemis*−/−^ cell line and the CJ reporter construct, an extrachromosomal V(D)J recombination assay, were employed to assess the activity of the above Artemis mutants. Mechanistically, upon cleavage of recombination signal sequences (RSSs) in the pCJ substrate by mouse RAG1/2 complex, the DNA hairpin ends will be opened by Artemis and then the dsDNA breaks can be ligated by the NHEJ repair mechanism. When functional Artemis was introduced into 293T^*Artemis*−/−^ cells, successful DNA repair enables GFP expression, which is then quantified by flow cytometry in 293T^*Artemis–/–*^ cells (Fig. 1b). Using this reporter system, we found that the G6E, E10G, F19L, and L110R mutations markedly impaired Artemis activity ex vivo (Fig. 1c). To further investigate the activity of these Artemis mutants, we prepared three DNA substrates for in vitro activity assays (Fig. 1d). As shown in Fig. 1e, all four Artemis mutants exhibited reduced exonuclease activity when incubated with single-stranded DNA. Consistently, the endonuclease activity of these mutants was also markedly impaired, as they displayed limited activity toward 3′ DNA overhang substrates and DNA hairpin substrates (Fig. 1f, g). Together, we newly identified that G6E, E10G, F19L, and L110R mutations in Artemis severely impair both its exonuclease and endonuclease activities, thereby potentially leading to ART-SCID. All mutations documented in previous reports and this study are all listed in Table S1.

### Three SCID-associated mutations in Artemis are chosen for editing by CBEs and ABEs

Although numerous mutations across the *Artemis* gene have been linked to loss of enzymatic activity and the development of SCID, only a subset of variants are amenable to be corrected by base editing. To systematically identify editable residues, we performed an additional analysis of those pathogenic mutations listed in Table S1, and those that could, in principle, be corrected using cytosine base editors (CBEs), adenine base editors (ABEs), DAF-CBE2, or DAF-TBE2, which mediate C-to-T, A-to-G, C-to-G, and T-to-G conversions, respectively, were selected to presented in Table 1 (Gaudelli et al. 2017; Komor et al. 2016; Ye et al. 2024). Table 1 summarized the potentially editable mutations, together with their associated activities, predicted sgRNAs, bystander edits, and PAM requirements.

**Table 1:**
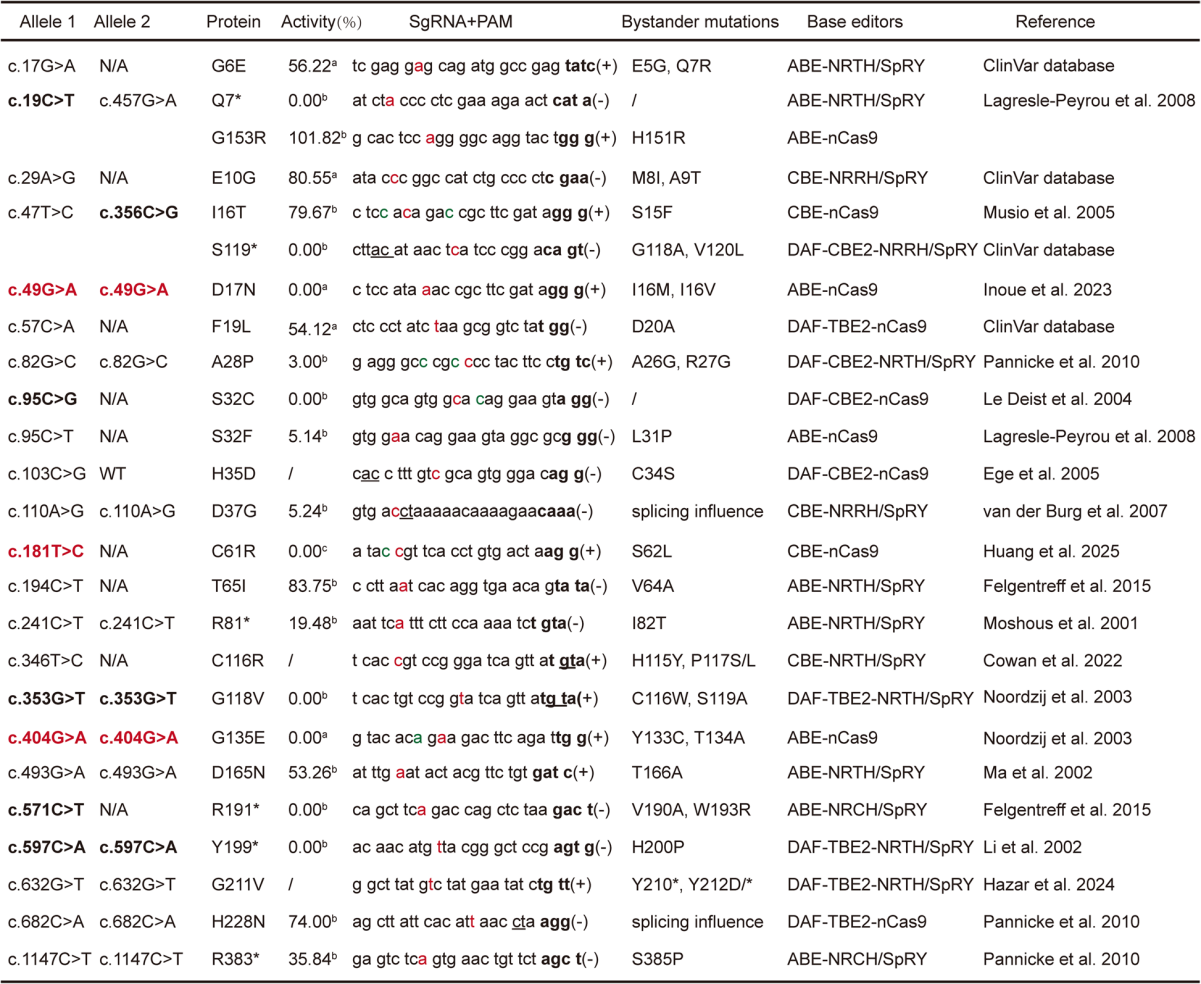
Artemis mutations suitable for correction by base editors. Alleles shown in bold represent loss-of-function mutations, and those highlighted in red indicate mutations targeted for correction by base editors in this study. In the sgRNA + PAM column, nucleotides in red indicate the target site, while bolded nucleotides represent the PAM sequence. Nucleotides in green indicate base editing does not alter the encoded amino acid due to codon degeneracy. Underlined nucleotides denote splice donor sites. a, Data from this study indicated the level of extrachromosomal V(D)J recombination activity (%) of Artemis mutants. b, data were obtained from Felgentreff et al. 2015 (Felgentreff et al. 2015). c, data from Huang et al. 2025 (Huang et al. 2025). A plus sign (+) indicates editing occurs on the coding strand, and a minus sign (–) indicates editing on the non-coding strand. Potential bystander mutations were predicted based on the specific base editor. For ABEs and CBEs, nucleotides within positions +2 to +10 relative to the protospacer were considered susceptible to editing, while positions +7 to +15 were predicted to be affected when using DAF-CBE2 and DAF-TBE2. N/A, not applicable; indicates that allelic information was unavailable or that the mutation was not a point mutation

**Fig. 2:**
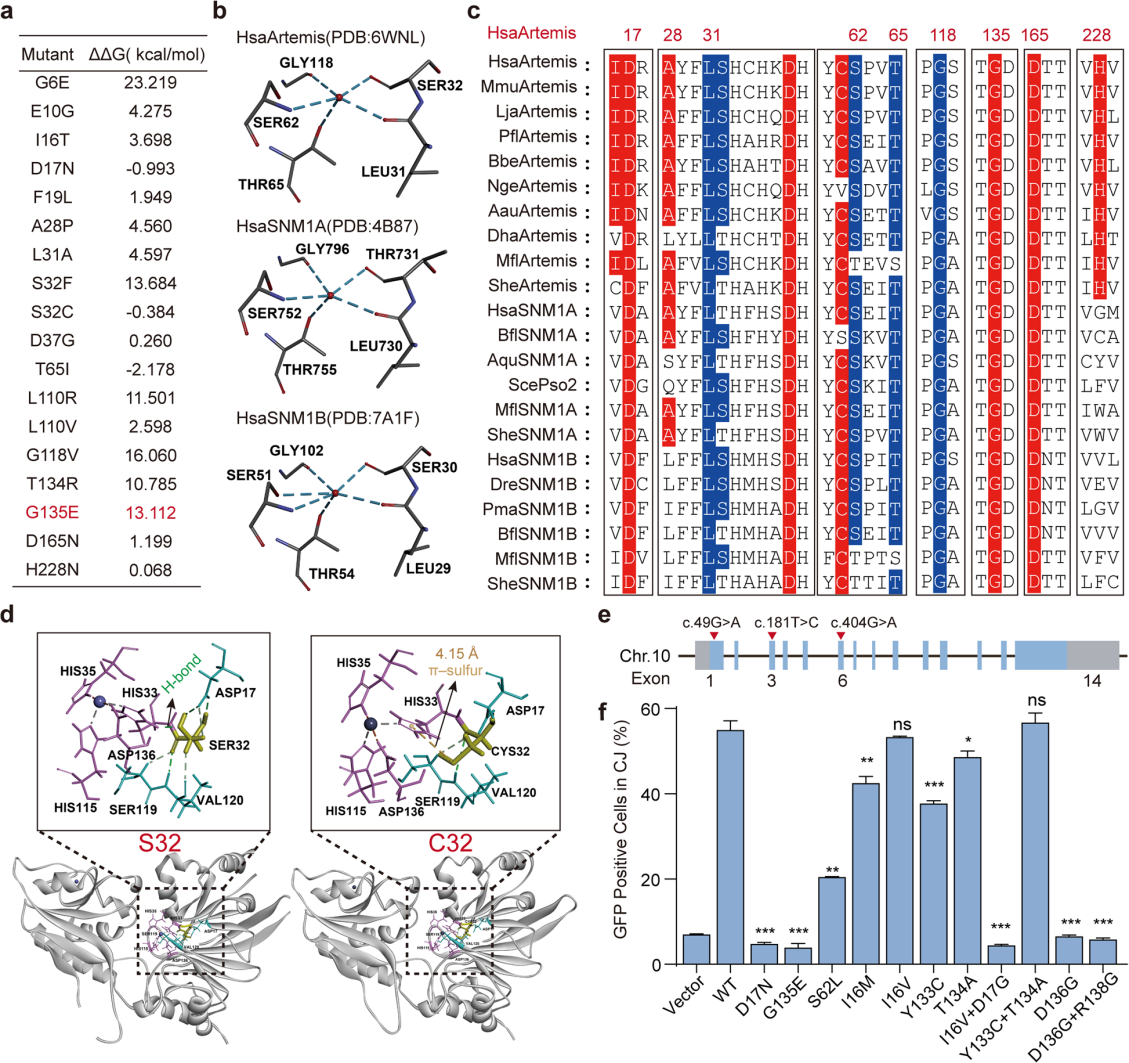
Three Artemis mutations are amenable to correction by base editors. **a** The free energy of Artemis mutants was calculated using Aggrescan3D (https://biocomp.chem.uw.edu.pl/A3D2/). The G135E mutant selected for editing exhibits improved free energy. **b** Structural modelling of Artemis, SNM1A, and SNM1B revealed that five conserved residues are capable of forming hydrogen bonds with a shared water molecule. **c** Multiple sequence alignment revealed the disease-causing amino acid mutations with evolutionary conservation. Hsa, *Homo sapiens*; Mmu, *Mus musculus*; Lja, *Lampetra japonica*; Pfl, *Ptychodera flava*; Bbe, *Branchiostoma belcheri*; Nge, *Notospermus geniculatus*; Aau, *Aurelia aurita*; Dha, *Debaryomyces hansenii*; Mfl, *Mylnosiga fluctuans*; She, *Salpingoeca helianthica*; Bfl, *Branchiostoma floridae*; Aqu, *Amphimedon queenslandica*; Sce, *Saccharomyces cerevisiae*; Dre, *Danio rerio*; Pma, *Petromyzon marinus*. **d** S32C mutation in Artemis disrupts the original hydrogen bond between S32 and H33, replacing it with a π–sulfur interaction between C32 and H33. **e** Locations of the c.49G > A, c.404G > A, and c.181T > C mutations within the *Artemis* gene. **f** CJ reporter assays indicated that the bystander mutations associated with editing of c.49G > A, c.404G > A, and c.181T > C have functional consequences. Flow cytometry results are presented as mean ± SD from three biological replicates. *P* values were calculated using Student’s t-test. ns: not significant

Based on above analysis, we subsequently focused on variants highlighted in bold in Table 1 that completely abolish Artemis activity. These mutations included Q7*, S119*, D17N, S32C, C61R, G118V, G135E, R191*, and Y199*. Among these mutations, Q7*, S119*, R191* and Y199* are premature stop mutations occurring in the catalytic domain, while structural impairment of c.49G > A (D17N) and c.181T > C (C61R) mutations in Artemis have been reported previously (Huang et al. 2025; Poinsignon et al. 2004). To obtain more information about the remaining missense mutations, we performed further structural analysis using SWISS-MODEL. We modeled the mutated proteins based on the wild-type structure (PDB: 6WNL) and calculated relative free energies. Results showed that, the G118V, G135E, and L110R mutations substantially reduced protein stability (Fig. 2a). Moreover, G118 coordinates a conserved water molecule that bridges L31, S32, S62, and T65, all of which have been reported to be critical for Artemis activity (Table 1) (Huang et al. 2025). This interaction network is conserved in the related SNM family members SNM1A and SNM1B (PDB: 4B87 and 7A1F), highlighting the catalytic and evolutionary importance of these five residues (Fig. 2b, c). The S32C substitution disrupts the S32-H33 hydrogen bond and introduces a π-sulfur interaction near key catalytic residues (H33, H35, H115, D136), likely inducing conformational changes that impair enzymatic activity (Fig. 2d).

Based on functional and structural analyses of these loss-of-function mutations, we next screened for homozygous mutations that could be efficiently targeted by ABEs or CBEs, given their high editing efficiencies and established clinical applicability. c.49G > A (D17N) and c.404G > A (G135E) were then identified as amenable to ABE-mediated correction. In addition, we included c.181T > C (C61R) as a representative CBE-editable mutation to explore the feasibility of cytosine base editing (Table 1). The c.49G > A mutation was identified in a Japanese SCID study, which included eight patients, two of whom harbored homozygous c.49G > A mutations. These patients were diagnosed before six months of age, presenting with severe infections and profound T cell deficiency; one also exhibited skin erythema (Inoue et al. 2023). The c.404G > A mutation was reported in another study, which included four ART-SCID patients. Two of these patients carried homozygous c.404G > A mutations, both exhibiting a T⁻B⁻NK⁺ immunophenotype. Clinically, one patient presented with persistent varicella-zoster virus infection at diagnosis, and both ultimately succumbed to severe disease complications, including multiorgan failure (Noordzij et al. 2003). Notably, these mutations are distributed across different exons, with c.49G > A located in exon 1, c.181T > C in exon 3, and c.404G > A in exon 6 (Fig. 2e).

Finally, to ensure the safety of editing these three mutations, adenines and cytosines at positions + 2 to + 10 of the protospacer were assessed for potential bystander effects. For ABE-mediated correction of c.49G > A and c.404G > A, predicted single bystander substitutions included I16M, I16V, Y133C, and T134A. For CBE-mediated correction of c.181T > C, S62L was predicted (Table 1). Functional evaluation in 293T^*Artemis–/–*^ cells showed that S62L attenuated but did not abolish activity, I16M and Y133C caused minor reductions, T134A had negligible impact, and I16V had no detectable effect (Fig. 2f). Collectively, these results demonstrate that c.49G > A, c.404G > A, and c.181T > C are suitable and safe targets for base editing, with minimal functional consequences from predicted bystander edits.

### Editing *Artemis* c.181T > C mutation ex vivo by CBEs

**Fig. 3:**
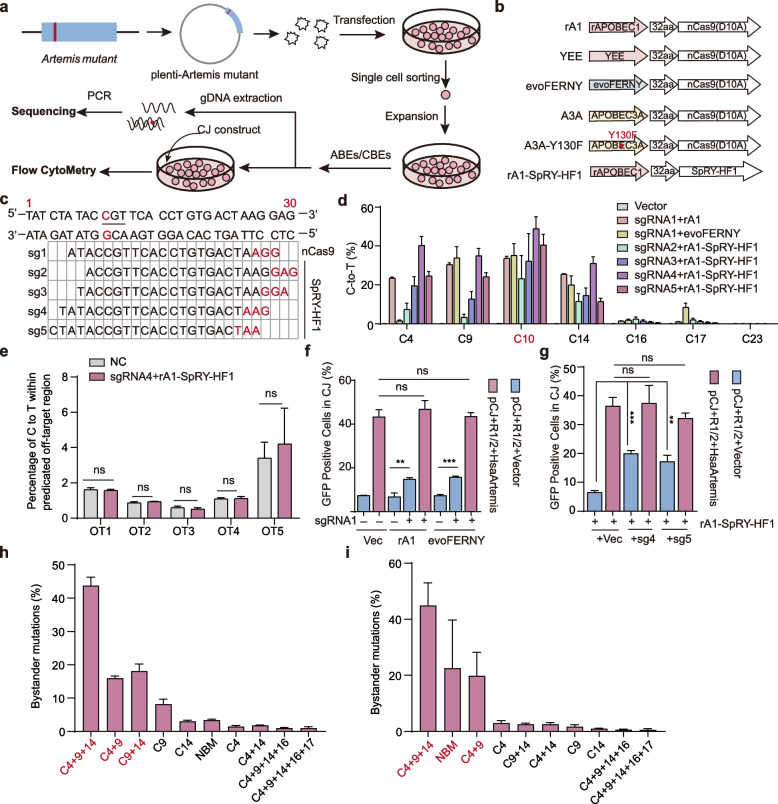
The *Artemis* c.181T > C mutation was efficiently corrected by CBEs. **a** Workflow for the generation of 293T^*T181C*^, 293T^*G49A*^ and 293T^*G404A*^ cell lines and assessment of editing efficiency by CBEs or ABEs. Human *Artemis* gene fragments carrying either the c.181T > C, c.49G > A or c.404G > A mutation were packaged into lentiviral vectors and transduced into 293T^*Artemis*–/–^ cells. After puromycin selection, single-cell clones were isolated for further experiments. **b** Various cytidine deaminases were incorporated into the BE4max construct in combination with nCas9 or SpRY-HF1. **c** Various sgRNAs were designed for editing using different base editors. **d** Quantitative analysis of editing outcomes of base editors at the target site and bystanders using deep sequencing. **e** rA1-SpRY-HF1-BE4 exhibited no significant sgRNA4-dependent off-target effects when editing the *Artemis* c.181T > C mutation. NC, the negative control, indicates that no base editor was transfected. **f** Artemis endonuclease activity was significantly increased following base editing by rA1-BE4 or evoFERNY-BE4 in 293T^*T181C*^ cells with sgRNA1. **g** Artemis endonuclease activity was significantly increased following rA1-SpRY-HF1 editing using sgRNA4 and sgRNA5 in 293T^*T181C*^ cells. **h**-**i** Bystander mutation frequency within successfully edited c.181C > T alleles mediated by sgRNA1 (**h**) and sgRNA4 (**i**). Red bases represent predominant combinatorial bystander mutations. NBM: No Bystander Mutation. All results are presented as mean ± SD from three biological replicates. *P* values were calculated using Student’s t-test. ns: Not significant

To enable base editing of *Artemis* mutations, we established an ex vivo pipeline (Fig. 3a). First, *Artemis* mutants were transduced into 293T^*Artemis*–/–^ cells using lentiviral vectors, followed by single-cell sorting and clonal expansion. Next, cells expressing the *Artemis* mutants (293T^*T181C*^, 293T^*G404A*^ and 293T^*G49A*^) were transfected with CBE or ABE constructs and incubated for three days. A portion of the cells was collected for sequencing, while the remainder was used to assess activity of the corrected Artemis.

To edit the c.181T > C mutation, we employed several high-activity cytidine deaminases, including rat APOBEC1 (rA1), APOBEC3A (A3A), and evoFERNY, which were incorporated into the BE4max backbone, a base editor known for its high editing efficiency (Fig. 3b). Given that the S62L bystander mutation may affect Artemis activity, narrow-window base editor variants, such as YEE and A3A-Y130F, were also tested to enable more precise editing (Kim et al. 2017; Koblan et al. 2018; Thuronyi et al. 2019; Wang et al. 2018) (Fig. 3b). In addition, rA1 was combined with SpRY-HF1, a PAM-relaxed Cas9 variant with high fidelity (Walton et al. 2020), to further expand sgRNA design options in editing assays (Fig. 3b). We next designed multiple sgRNAs, including one with the target nucleotide positioned at + 5 relative to the protospacer and a downstream NGG PAM (sgRNA1; Fig. 3c). In addition, other sgRNAs were designed with the target site located between positions + 3 and + 7 relative to the protospacer and a downstream NRN PAM, enabling editing using rA1-SpRY-HF1 (Fig. 3c).

We next transfected 293T^*T181C*^ cells with different CBE constructs and sgRNAs. Sanger sequencing revealed that, when guided by sgRNA1, both rA1-BE4max and evoFERNY-BE4max exhibited higher editing efficiency at the target site than A3A-BE4max (Fig. S1a), whereas YEE-BE4max showed no detectable editing activity (Fig. S1a). In addition, rA1-SpRY-HF1 displayed robust editing efficiency when paired with sgRNA4 or sgRNA5 (Fig. S1a). Deep sequencing further confirmed that rA1-BE4max and evoFERNY-BE4max achieved correction rates of approximately 30% without substantial off-target effects at the predicated sites compared with the control (Fig. 3d, S1b). Specifically, off-target sites were predicted based on the sgRNA sequences using Cas-OFFinder, and five predicted sites for each sgRNA were selected for experimental analysis. Targeted deep sequencing was performed, and CRISPResso2 was used to calculate on-target and off-target editing frequencies. The off-target editing efficiency at each site was determined by calculating the total frequency C-to-T conversions within the sgRNA target region. Notably, rA1-SpRY-HF1 combined with sgRNA4 reached ~ 50% editing efficiency at the target site and similarly showed no significant off-target activity at any of the computationally predicted genomic loci (Fig. 3d, e). Importantly, flow cytometry analyses demonstrated that editing mediated by rA1-BE4max, evoFERNY-BE4max, or rA1-SpRY-HF1 partially restored Artemis activity in 293T^*T181C*^ cells (Fig. 3f, g). We also found that bystander effects were relatively prominent during c.181T > C editing. To further investigate the impact of bystander mutations on editing outcomes, we re-analyzed our deep sequencing data by filtering for sequences harboring successful edits at the target sites. This haplotype-level analysis enabled us to quantify the allelic co-occurrence frequency of intended modifications and bystander mutations within the same allele. The results revealed that sites 4, 9, and 14 exhibited the highest frequencies of bystander mutations in both sgRNA1- and sgRNA4-guided editing (Fig. 3h, i). Substitutions at sites 4 (CTA to TTA, both encoding leucine) and 9 (TAC to TAT, both encoding tyrosine) do not alter the amino acid sequence due to codon degeneracy. Therefore, the predominant functional bystander effect is attributable to mutations at site 14, which result in an S62L substitution. As shown in Fig. 2f, the S62L mutation alone leads to a moderate reduction in Artemis activity. Thus, the c.181T > C mutation could be efficiently corrected using CBE editors, and such editing could partially restore the activity of Artemis.

### Ex vivo correction of *Artemis* c.49G > A and c.404G > A mutations using ABEs

**Fig. 4:**
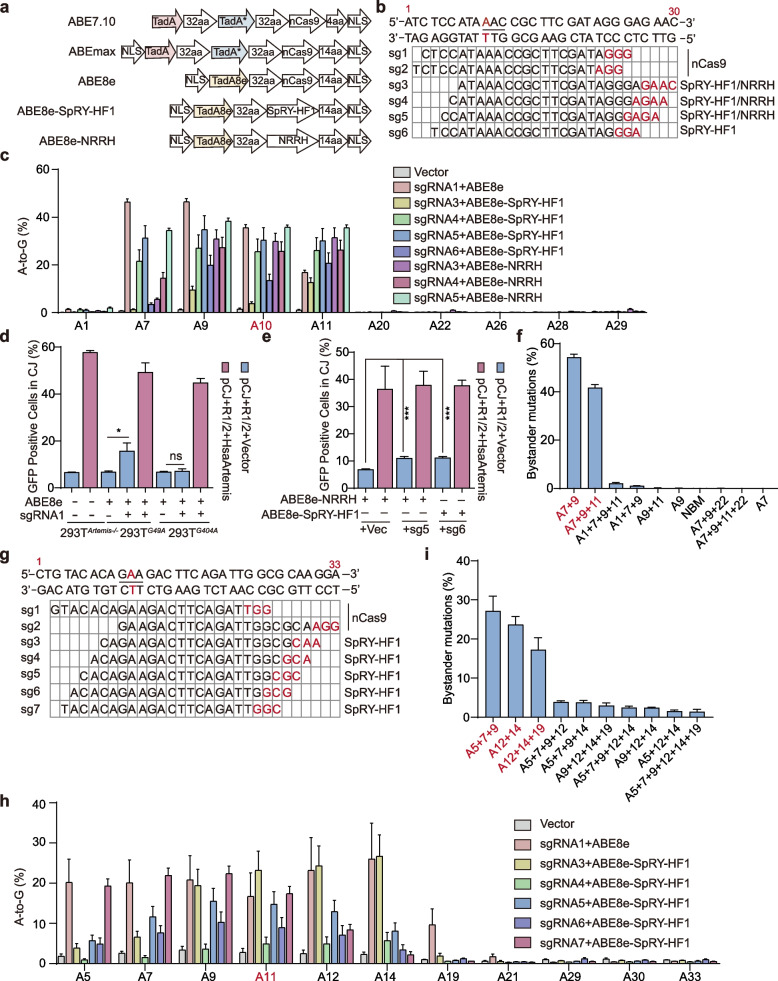
Ex vivo editing of the *Artemis* c.49G > A mutation was efficiently achieved using ABE8e. **a** Five base editors were used for editing c.49G > A and c.404G > A mutations ex vivo. **b** Various sgRNAs were designed for editing c.49G > A mutation. **c** Both ABE8e and ABE8e-NRRH achieved approximately 35% editing efficiency at the c.49G > A site. **d** ABE8e-mediated correction of the c.49G > A mutation significantly enhanced Artemis activity ex vivo, whereas correction of the c.404G > A mutation failed to restore activity. **e** Correction of the c.49G > A mutation by ABE8e-SpRY-HF1 and ABE8e-NRRH significantly enhanced Artemis activity ex vivo. **f** Bystander mutation frequency within successfully edited alleles of c.49G > A mediated by sgRNA1. Red bases represent predominant combinatorial bystander mutations. **g** Various sgRNAs were designed for editing c.404G > A mutation. NC, the negative control, indicates that no base editor was transfected. **h** Both ABE8e and ABE8e-SpRY-HF1 achieved approximately 20% editing efficiency at the c.404G > A site. **i** Bystander mutation frequency within successfully edited alleles of c.404G > A mediated by sgRNA1. NBM: No Bystander Mutation. All results are presented as mean ± SD from three biological replicates. *P* values were calculated using Student’s t-test. ns: Not significant

To correct the *Artemis* c.49G > A and c.404G > A mutations, we first tested five adenine base editors, including ABE7.10, ABEmax, ABE8e, ABE8e-SpRY-HF1, and ABE8e-NRRH, the latter two incorporating PAM-relaxed Cas9 variants (Fig. 4a) (Thuronyi et al. 2019; Walton et al. 2020). For c.49G > A, six sgRNAs were designed, with sgRNA1 and sgRNA2 compatible with canonical NGG PAMs, while the remaining four were targetable by SpRY-HF1 or NRRH (Fig. 4b).

Upon transfection into 293T^*G49A*^ cells, Sanger sequencing revealed that ABE8e combined with sgRNA1 induced substantial editing at the target site (Fig. S2a). ABE7.10 and ABEmax showed no detectable on-target editing but produced notable bystander edits at adjacent positions. Interestingly, sgRNA2 which differs from sgRNA1 by a single nucleotide failed to support target editing with any editor, though bystander edits were again observed (Fig. S2a). Among the PAM-relaxed sgRNAs, ABE8e-NRRH paired with sgRNA5 exhibited the highest efficiency, while the others showed variable activity (Fig. S2b). Deep sequencing confirmed that ABE8e with sgRNA1 achieved ~ 35% correction efficiency, comparable to ABE8e-NRRH with sgRNA5 (Fig. 4c). Flow cytometry using the CJ reporter assay demonstrated that ABE8e-, ABE8e-NRRH–, and ABE8e-SpRY-HF1 mediated editing significantly increased Artemis activity in 293T^*G49A*^ when guided by sgRNA1, sgRNA5 and sgRNA6, respectively (Fig. 4d, e).

Importantly, we found no evidence of significant off-target mutations at the predicted genomic sites, confirming the precision of our sgRNA1 design (Fig. S3a). In c.49G > A editing with sgRNA1, the most frequent (~ 55%) bystander mutations occur at sites 7 and 9 (Fig. 4f). Notably, the resulting I16V substitution does not significantly alter Artemis activity compared with the wild-type protein (Fig. 2f). This finding is consistent with our CJ reporter assay, which shows that correction of c.49G > A can partially rescue Artemis activity (Fig. 4d).

To correct the c.404G > A mutation, we designed seven sgRNAs, with sgRNA1 and sgRNA2 compatible with canonical NGG PAMs and the remaining five targetable by SpRY-HF1 (Fig. 4g). Using the same editing pipeline, distinct ABEs were transfected into 293T^*G404A*^ cells. For canonical PAM sgRNAs, Sanger sequencing revealed that only ABE8e paired with sgRNA1 efficiently edited the target site (Fig. S2c). Among the PAM-relaxed sgRNAs, sgRNA3 exhibited the highest editing efficiency, while the others showed variable activity (Fig. S2d). Next-generation sequencing confirmed that ABE8e with sgRNA1 and ABE8e-SpRY with sgRNA3 achieved comparable editing efficiencies of ~ 20% (Fig. 4h). Furthermore, our analysis of the predicted in silico off-target sites revealed that sgRNA1 did not induce significant off-target mutations (Fig. S3b).

However, ABE8e-mediated editing at c.404G > A with sgRNA1 did not increase Artemis activity (Fig. 4d). For c.404G > A editing with sgRNA1, the most frequent bystander mutations involve concurrent substitutions at sites 5, 7, and 9. The second most frequent pattern occurs at sites 12 and 14, followed by mutations at sites 12, 14, and 19 (Fig. 4i). These mutation patterns correspond to Y133C + T134A, D136G, and D136G + R138G substitutions, respectively. We found that the Y133C + T134A mutations do not affect Artemis activity, whereas either D136G alone or the combined D136G + R138G mutations completely abolish its activity (Fig. 2f). These results suggest that the predominant D136G bystander mutation may account for the failure of c.404G > A editing to restore function. Overall, these results highlight that ABEs efficiently edits the Artemis c.49G > A mutation ex vivo.

## Discussion

Artemis mutations arrest T and B cell development by disrupting V(D)J recombination and DNA double-strand break repair, causing RS-SCID. In this study, we not only summarized activity-impairing mutations but also identified several previously uncharacterized functional residues of Artemis. Furthermore, three mutated sites, the c.49G > A, c.181T > C, and c.404G > A, were chosen to evaluate the therapeutic potential of base editors for ART-SCID. Our results showed that rAPOBEC1-SpRY-HF1-BE4max achieved ~ 50% editing efficiency at the c.181T > C site, while ABE8e reached ~ 35% and ~ 20% editing efficiency at the c.49G > A and c.404G > A sites, respectively. In all, these findings provide proof-of-concept that base editing can correct ART-SCID-associated mutations, highlighting its potential as a therapeutic strategy for Artemis single-nucleotide variants, particularly when bystander edits do not affect key catalytic residues and thus preserve enzyme activity. However, when deleterious bystander effects occur, the use of base editors with a more constrained editing window may be required to minimize unwanted substitutions and improve therapeutic safety and efficacy.

However, there are still some limitations about this study. First, we only tested A-to-G and C-to-T conversions using ABEs and CBEs, two widely used types of base editors. However, other types of mutations also occur in Artemis, such as C-to-G and G-to-T (Table 1). Beyond the conventional A-to-G and C-to-T conversions, recent advances in DNA glycosylase-mediated base editing have substantially broadened the nucleotide substitution repertoire, now encompassing C-to-G, T-to-C, T-to-G, G-to-T, and G-to-C conversions (Tong et al. 2023, 2024; Ye et al. 2024). Building on these advances, many *Artemis* mutations amenable to be corrected by DNA glycosylase-based editors in combination with Cas9 variants recognizing non-canonical PAM sequences should be candidates for future therapeutic testing (Table 1), which will further expand the applicability of base editing for ART-SCID and other monogenic disorders. In addition, it is important to note that base editing is not a viable strategy for patients harboring homozygous large genomic deletions, as these cases lack a targetable template for nucleotide conversion. The therapeutic rationale for correcting a single allele in compound heterozygous patients is supported by clinical inheritance patterns. Literature demonstrates that while biallelic mutations drive SCID manifestations, parents carrying only a single mutant allele remain asymptomatic (Inoue et al. 2023; Strubbe et al. 2021; Sundin et al. 2019; Volk et al. 2015). For instance, Inoue et al. described a patient with a homozygous exon 1—3 defect whose monoallelic parents were healthy. This confirms that if a patient harbors a large deletion alongside a targetable point mutation, correcting just the point allele would effectively convert their genotype to a carrier state.

Second, although editing of the c.404G > A mutation achieved ~ 20% efficiency, Artemis activity was not significantly restored. This is likely due to a bystander mutation at D136 (Fig. 4i), a residue that may be important for Artemis activity (Poinsignon et al. 2004). Alternative gene-editing approaches, such as CRISPR-Cas9 mediated HDR and prime editing (PE), may provide more suitable strategies for correcting these types of mutation. Prime editing enables precise nucleotide correction by coupling Cas9 with a reverse transcriptase and using an exogenous RNA template, thereby markedly reducing bystander editing effects (Anzalone et al. 2019). After multiple rounds of optimization, the efficiency of prime editing can reach ~ 50% in optimized settings or at certain loci and in specific cell types (Yan et al. 2024). Although its efficiency in cells remains lower than that of base editing, its precision makes it a promising tool for future gene therapy applications (Chen et al. 2021; Chen and Liu 2023).

Base editing therapy has now progressed to the clinical development stage, with multiple trials underway for diseases such as SCD, β-thalassemia, familial hypercholesterolemia, and universal CAR-T therapy (Jiang and Yang 2023). Preclinical studies in β-thalassemia and SCID have demonstrated durable therapeutic editing in self-renewing HSPCs using patient-derived CD34⁺ cells (Liao et al. 2023; McAuley et al. 2023). All editing experiments in this study were performed in 293T cells, which provide a convenient and robust system for initial evaluation but do not fully recapitulate the biology of human HSPCs. Unlike 293T cells, primary CD34 + HSPCs exhibit distinct DNA repair pathway preferences and are more sensitive to the cytotoxicity associated with genome editing components (Shin et al. 2020). Future studies on CD34 + HSPCs may require clinically relevant delivery methods, such as electroporation of ribonucleoprotein (RNP) complexes, which exhibited a good balance between cytotoxicity and efficiency of genomic rearrangements as compared to plasmid-based delivery (Lattanzi et al. 2019). Furthermore, maintaining the stemness and multi-lineage potential of HSPCs requires specialized culture optimization, including the use of specific cytokines or small molecules to prevent premature differentiation during the editing process. Most critically, the therapeutic efficacy and safety of base-edited HSPCs must be validated through long-term engraftment assays in immunodeficient mouse models to ensure that the edited cells retain their self-renewal capacity and do not undergo malignant transformation. In addition, our off-target analysis was limited to predicted sites; therefore, more comprehensive, genome-wide evaluation in HSPCs is required in future studies to fully define the precision of this approach. These considerations are essential for establishing the realistic therapeutic potential of base editing for ART-SCID.

Nevertheless, despite the preliminary nature of this study and the use of a simplified cellular model, our findings provide clear proof-of-concept that base editing can be leveraged to correct pathogenic Artemis mutations associated with ART-SCID. By linking precise nucleotide correction to functional outcomes, this work highlights the therapeutic relevance of base editors, particularly for mutations amenable to efficient and accurate editing. Although further optimization will be required to improve precision, reduce bystander effects, and extend these approaches to clinically relevant cell types such as patient-derived CD34^+^ HSPCs, the continued rapid development of base and prime editing technologies offers strong potential for clinical translation. Collectively, our results expand the functional understanding of Artemis mutations and establish a theoretical foundation for future gene-editing–based therapies for ART-SCID and related monogenic disorders.

## Materials and methods

### Cells

The 293T cell lines from ATCC (Cat# CRL-3216, RRID: CVCL_0063) were maintained in DMEM (cat.: C11995500BT, Gibco) supplemented with 10% FBS at 37 °C under 5% CO_2_. 293T cells deficient in Artemis (293T^*Artemis*−/−^) were generated in a previous study (Huang et al. 2025). Transfections were performed using jetPRIME (cat.: 114–15, PolyPlus-transfection Bioparc.) according to the manufacturer’s instructions.

### Fluorescent reporter assays

293T ^*Artemis−/−*^ cells in 24-well plates were cultured and transfected with the CJ construct vectors (pCJ, mouse RAG1 and mouse RAG2) and indicated Artemis protein expression vectors per well by jetPRIME. After 4 h transfection, culture was replaced. After 48 h, cells were digested with trypsin and collected by centrifugation, then washed once with PBS and resuspended in PBS. GFP expression was analyzed using a Beckman FACS Calibur.

### Plasmids construction

Human *Artemis* genes were constructed into the pEZ-Flag (cat.: EX-NEG-M12, iGene Biotechnology) vectors for protein expression. *Artemis* gene mutants were prepared by QuikChange Lightning Multi Site-Directed Mutagenesis Kit (cat.: #210513, Agilent). The SpRY-HF1 gene was cloned from pCAG-CBE4max-SpRY-P2A-EGFP (Addgene plasmid #139999). The NRRH gene was generated by introducing mutations into the nCas9.

### Protein purification

Expi293F cells were maintained in Hi-exp 293 medium (cat.: AC601501, OPM Bioscience) at 37 °C in an 8% CO₂ atmosphere. For protein expression, the cells were transfected with the pEZ-Artemis plasmid using polyethylenimine (PEI) as the transfection reagent. The DNA and PEI were mixed at a ratio of 1:4 (w/w) before being added to the cell culture. The cells were collected after transfection with indicated vectors for 96 h and washed twice by pre-cold PBS. Cells were lysed with lysis buffer (1% TritonX-100, 25 mM Tris (pH 7.4), 500 mM NaCl, 2 mM EDTA and 1 × protease inhibitor cocktail (cat.: AG21501, Accurate Biology)). After centrifugation with 12,000 g for 10 min at 4 °C, the cell lysis supernatants were immunoprecipitated with anti-Flag resins (cat.: P9801, Beyotime Biotechnology) for 3 h at 4 °C. The resins were slowly washed five times with wash buffer (1% TritonX-100, 25 mM Tris (pH 7.4), 500 mM NaCl, 2 mM EDTA and 1 mM PMSF). Then samples were eluted with elution buffer (25 mM Tris (pH 7.4), 500 mM NaCl, 1 mM DTT) containing 5 μg/μl 3 × Flag peptides (cat.: F4799, Sigma-Aldrich) according to manufacturer’s instructions and dialyzed with dialysis buffer (25 mM Tris (pH 7.4), 150 mM KCl, 10% glycerol) through ultra-filtration. The purified protein concentration was quantified using Detergent Compatible Bradford Protein Assay Kit (cat.: P0006C-1, Beyotime Biotechnology) and stored at − 80 °C.

### In vitro cleavage assay

DNA cleavage substrates (Huang et al. 2025) were labeled with Cy5 fluorophore. Cleavage reactions (10 μl) were performed in reaction buffer containing 25 mM Tris (pH 7.4), 10 mM KCl, 10 mM MnCl₂, 0.25 mM ATP, 0.5 mg/ml BSA, and 1 mM DTT, and included 100 nM DNA substrates and 150 nM Artemis protein. Reactions were incubated at 37 °C for 2 h for DNA hairpin substrates or 1 h for ssDNA and DNA overhang substrates. Reactions were terminated by the addition of 10 μl stop buffer (98% formamide, 2% EDTA) and heated at 95 °C for 5 min. Samples were then resolved on 20% denaturing urea–TBE polyacrylamide gels. Following electrophoresis, gels were imaged using a Bio-Rad QualityOne system.

### Gene editing using the base editor systems

*Artemis* c.49G > A, c404G > A and c.181T > C mutant genes were integrated into the genomic DNAs of 293T^*Artemis−/−*^ cells using lentivirus vector. Each cytidine deaminase variant was individually cloned into BE4max construct. ABE and CBE constructs were then co-transfected with the pUC19-gRNA plasmid into 293T ^*G49A*^, 293T ^*G404A*^ and 293T ^*T181C*^ cells, respectively. In each well of a 6-well plate, 4 μg of plasmid DNA was used, with a 3:1 ratio of CBE or ABE to gRNA. After transfection for 3 days, we extracted genomic DNAs using DNeasy Blood & Tissue Kit (cat.: 175042784, QIAGEN) and designed primers to clone the target and off-target sequences. PCR products were purified using QIAquick PCR Purification Kit (cat.: 28106, QIAGEN) for Sanger sequencing and deep sequencing. All primers used for cloning the on-target and off-target sites are listed in Table S2.

### Off target and bystander mutation analysis

We used Cas OFFinder (http://www.rgenome.net/cas-offinder) to predict off-target sites and selected 5 predicted sites for further analysis (Table S2). CRISPResso2 was used to calculate the on target and off target frequency. The off-target editing efficiency at each site was assessed by calculating the total frequency of A-to-G or C-to-T conversions within the sgRNA target region. To analyze bystander effects, we filtered the deep sequencing data for sequences with successful target-site edits. The top 10 bystander mutation combinations are shown.

### Structural prediction of Artemis

Structures of human Artemis mutants were modeled by submitting the corresponding sequences to the SWISS-MODEL server (https://swissmodel.expasy.org/), utilizing the human Artemis X-ray crystal structure (PDB: 6WNL) as a reference template. The generated models underwent energy minimization in Discovery Studio to refine their structures. Subsequently, electrostatic potential calculations and structural visualizations were conducted using PyMOL, using its default settings.

### Quantification and statistical analysis

In all cases, data were processed and plotted using GraphPad Prism 8 and error bars denote SD of three biological replicates and statistical significance was determined using Student’s* t* test, where significance was defined as *p < 0.05, **p < 0.01, ***p < 0.001.

## Supplementary Information

Supplementary Material 1: Fig. S1 SpRY-HF1 significantly increased the editing efficiency of the Artemis c.181 T> C mutation. a Editing efficiency of different CBEs at the target site in 293T^*T181C*^ cells were evaluated by Sanger sequencing. The red arrows indicate the edited target sites. b Both rA1-BE4max and evoFERNY-BE4max exhibited no significant sgRNA1-dependent off-target effects when editing the *Artemis* c.181T > C mutation. NC, the negative control, indicates that no base editor was transfected. All results are presented as mean ± SD from three biological replicates. *P* values were calculated using Student’s t-test. ns: not significant. Sanger sequencing was conducted in three independent replicates, and one representative result is shown.

Supplementary Material 2: Fig. S2 Correction of the c.49G > A and c.404G > A mutations by ABEs. a Sanger sequencing results revealed that ABE8e exhibited higher editing efficiency at the c.49G > A site compared with ABE7.10 and ABEmax. b Sanger sequencing analysis of c.49G > A editing efficiencies using different sgRNAs in combination with ABE8e-SpRY-HF1 or ABE8e-NRRH. c—d Sanger sequencing analysis of c.404G > A editing efficiencies using different sgRNAs in combination with ABE8e (c) and ABE8e-SpRY-HF1 (d). The red arrows indicate the edited target sites. Sanger sequencing was conducted in three independent replicates, and one representative result is shown.

Supplementary Material 3: Fig. S3 Off-target analysis of c.49G > A and c.404G > A via targeted deep sequencing. a ABE8e exhibited no significant sgRNA1-dependent off-target effects when editing the Artemis c.49G > A mutation. b ABE8e exhibited no significant sgRNA1-dependent off-target effects when editing the Artemis c.404G > A mutation. NC, the negative control.

Supplementary Material 4: Table S1 Artemis mutations identified from literature and ClinVar.

Supplementary Material 5: Table S2 Primers used in cloning the on-target and off-target sites.

## Data Availability

All data generated or analyzed during this study are included in this published article and its supplementary information files.
