# Supplementary figures and images for: Base editing of Artemis mutations ex vivo sheds light on gene therapy for Artemis-deficient SCID
Source: Adv Biotechnol (Singap). 2026 May 15;4(2):20. doi: 10.1007/s44307-026-00115-w (PMC13179409; doi:10.1007/s44307-026-00115-w)

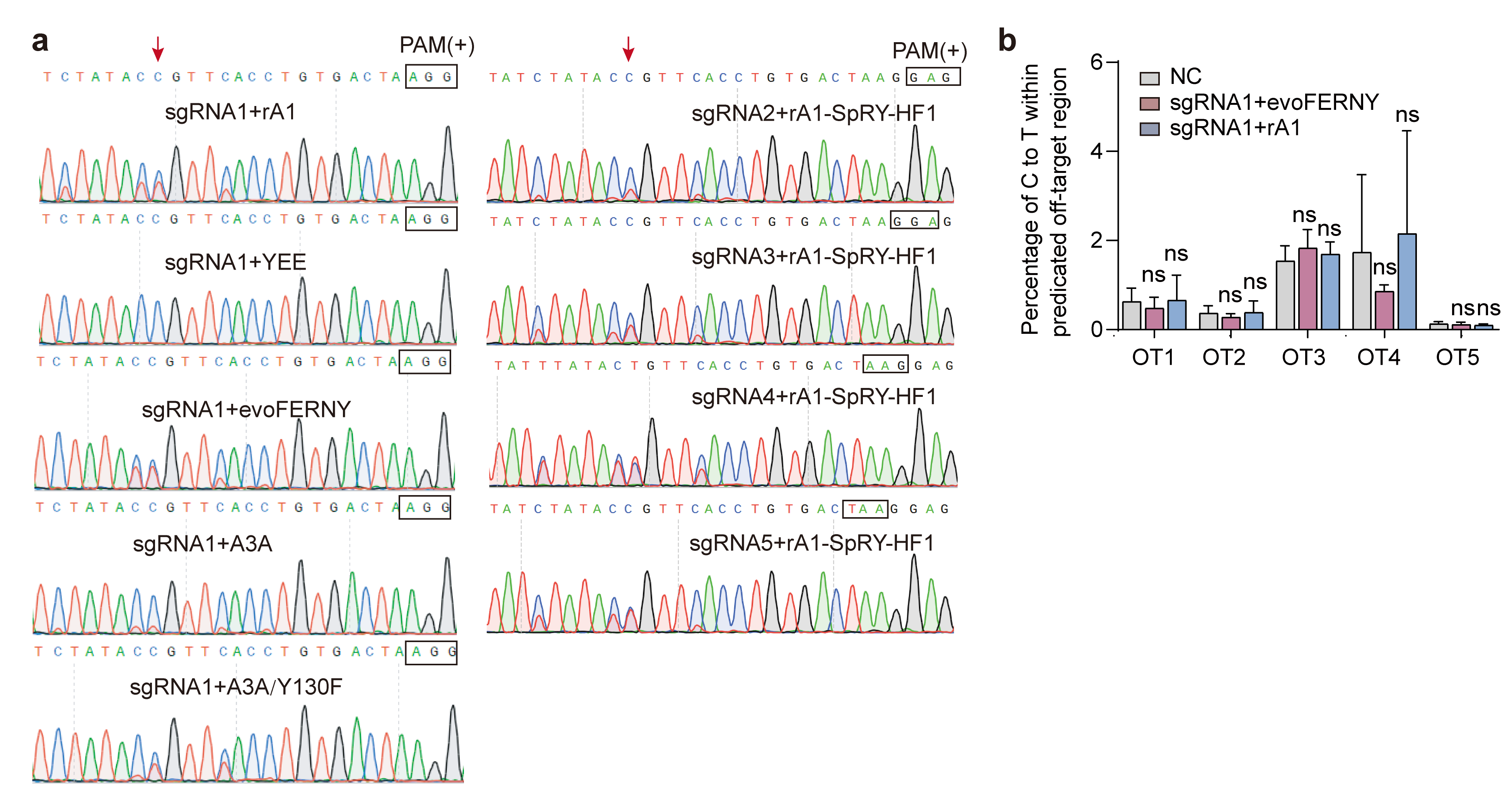

Supplement: Supplementary file 1 — Supplementary Material 1: Fig. S1 SpRY-HF1 significantly increased the editing efficiency of the Artemis c.181 T> C mutation. a Editing efficiency of different CBEs at the target site in 293TT181C cells were evaluated by Sanger sequencing. The red arrows indicate the edited target sites. b Both rA1-BE4max and evoFERNY-BE4max exhibited no significant sgRNA1-dependent off-target effects when editing the Artemis c.181T > C mutation. NC, the negative control, indicates that no base editor was transfected. All results are presented as mean ± SD from three biological replicates. P values were calculated using Student’s t-test. ns: not significant. Sanger sequencing was conducted in three independent replicates, and one representative result is shown. [file 44307_2026_115_MOESM1_ESM.tif]

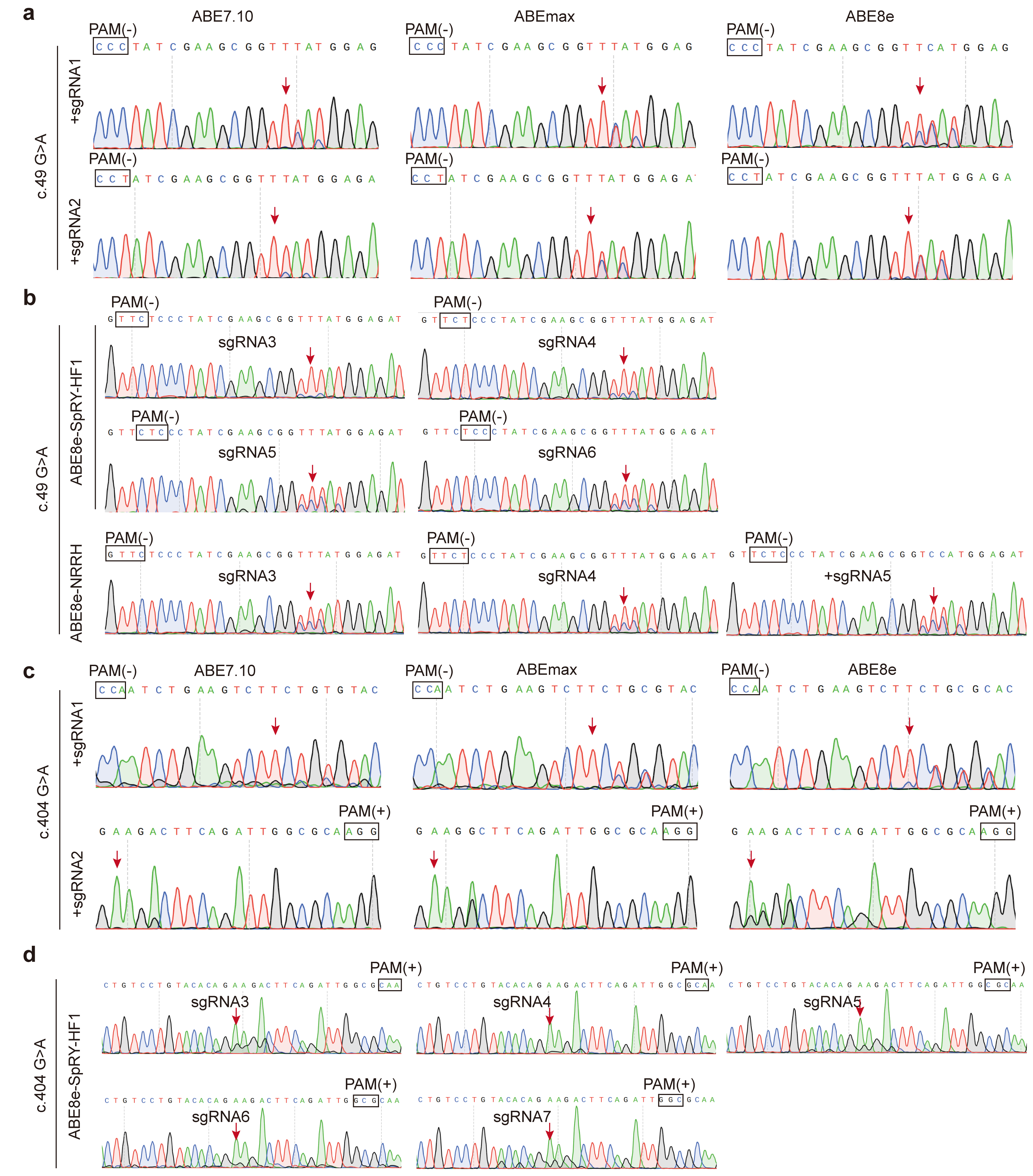

Supplement: Supplementary file 2 — Supplementary Material 2: Fig. S2 Correction of the c.49G > A and c.404G > A mutations by ABEs. a Sanger sequencing results revealed that ABE8e exhibited higher editing efficiency at the c.49G > A site compared with ABE7.10 and ABEmax. b Sanger sequencing analysis of c.49G > A editing efficiencies using different sgRNAs in combination with ABE8e-SpRY-HF1 or ABE8e-NRRH. c—d Sanger sequencing analysis of c.404G > A editing efficiencies using different sgRNAs in combination with ABE8e (c) and ABE8e-SpRY-HF1 (d). The red arrows indicate the edited target sites. Sanger sequencing was conducted in three independent replicates, and one representative result is shown. [file 44307_2026_115_MOESM2_ESM.tif]

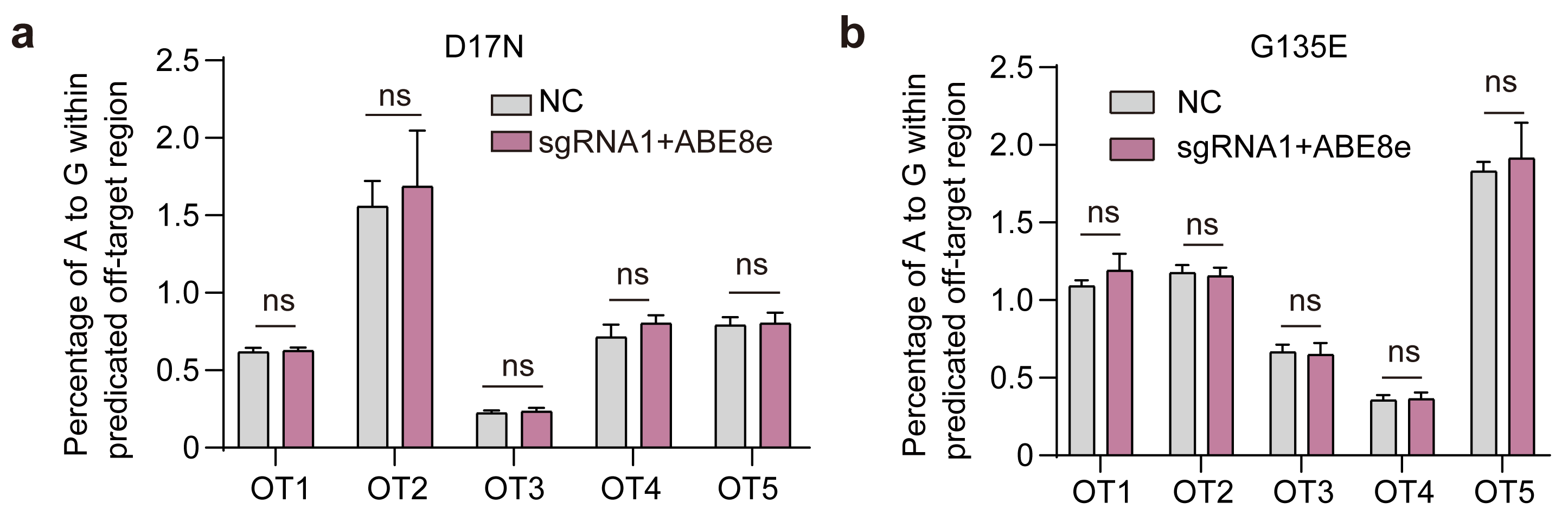

Supplement: Supplementary file 3 — Supplementary Material 3: Fig. S3 Off-target analysis of c.49G > A and c.404G > A via targeted deep sequencing. a ABE8e exhibited no significant sgRNA1-dependent off-target effects when editing the Artemis c.49G > A mutation. b ABE8e exhibited no significant sgRNA1-dependent off-target effects when editing the Artemis c.404G > A mutation. NC, the negative control. [file 44307_2026_115_MOESM3_ESM.tif]
